# Adding Adjuvants to Fluoropyrimidine-based Neoadjuvant Chemoradiotherapy for Locally Advanced Rectal Cancer: An Option Worthy of Serious Consideration

**DOI:** 10.7150/jca.48337

**Published:** 2021-01-01

**Authors:** Fengpeng Wu, Chaoxi Zhou, Bingyuan Wu, Xiaoxiao Zhang, Kanghua Wang, Jun Wang, Linlin Xiao, Guiying Wang

**Affiliations:** 1 Department of Radiation Oncology, Fourth Hospital of Hebei Medical University, Shijiazhuang, Hebei, China.; 2 Department of Colorectal Surgery, Fourth Hospital of Hebei Medical University, Shijiazhuang, Hebei, China.; 3 Department of Medical Imaging, Second Hospital of Hebei Medical University, Shijiazhuang, Hebei, China.

**Keywords:** locally advanced rectal cancer, neoadjuvant chemoradiotherapy, adjuvants, cytotoxic chemotherapeutic drugs, molecular targeted anticancer drugs, immune checkpoint inhibitors

## Abstract

The application of fluoropyrimidine-based neoadjuvant chemoradiotherapy (Fu-nCRT) of locally advanced rectal cancer (LARC) has become a common therapeutic regimen. In order to improve the efficacy and enable more patients to benefit from this treatment, an accumulation of studies have been carried out on the auxiliary use of other drugs with Fu-nCRT. However, due to specific challenges and the potential opportunities that coexist in this field, a more reasonable approach to the mode of treatment remains to be explored. In this review, we have summarized the results of the studies on the combination of Fu-nCRT with cytotoxic drugs, anti-tumor angiogenesis, and anti-EGFR agents, as well as the status of the application of immune checkpoint inhibitors in the neoadjuvant therapy of LARC patients.

## Introduction

At present, the standard treatment for locally advanced rectal cancer (LARC) is neoadjuvant therapy combined with total mesorectal excision (TME) surgery and postoperative systematic treatment 1,2, among which neoadjuvant therapy is of great significance for the selection of surgical type and subsequent treatment mode. The neoadjuvant treatment modalities recommended in the National Comprehensive Cancer Network (NCCN) 2 guidelines for LARC patients include long-course neoadjuvant chemoradiotherapy, namely fluoropyrimidine-based nCRT (Fu-nCRT), and short-course radiotherapy. Among them, Fu-nCRT is recommended for all LARC patients without contraindications to radiotherapy and chemotherapy, while short-course radiotherapy is not recommended for low-lying tumors less than 5 cm from the anus. Moreover, guidelines also emphasize the necessity of multidisciplinary assessments of tumor degradation and long-term toxicity of patients before applying short-course radiotherapy. Therefore, the adaptation of Fu-nCRT is more widely used in clinic than short-course radiotherapy, and it is considered to be the standard neoadjuvant treatment regimen for LARC patients.

Studies have found that neoadjuvant therapy can reduce tumor volume and degrade clinical staging in most patients, thereby improving the anal retention rate and reducing the local recurrence rate (LRR) 3,4. Despite this, the clinical response rate (cRR) and pathologic complete response (pCR) rate of patients still need to be improved. Current study results show that the cRR and pCR of LARC patients after Fu-nCRT treatment were 42-86% 5-8 and 11%-15% 9-10, and the overall survival (OS) rate was not significantly improved compared with postoperative adjuvant chemoradiotherapy 4,11. Considering that the chemotherapy drugs in the Fu-nCRT treatment mode contain only one type of fluorouracil, researchers conducted a large number of studies, hoping to improve the short-term/long-term efficacy of LARC patients by adding other anti-tumor drugs to the Fu-nCRT regimen. These antitumor agents include both traditional cytotoxic drugs and biological agents such as anti-vascular endothelial growth factor (VEGF) and anti-epidermal growth factor receptor (EGFR).

Our goal in this article is to summarize the short/long-term efficacy, therapeutic toxicity, and postoperative complications of adding antitumor drugs to the Fu-nCRT regimen for LARC patients, so as to provide a reference for clinicians to choose the preoperative nCRT regimen for LARC patients and carry out relevant studies.

## Cytotoxic chemotherapeutic drugs

Cytotoxic chemotherapeutic drugs can prevent cell division by preventing DNA synthesis and transcription as well as repairing damage, which results in cell death. The ability of cytotoxic drugs to kill cancer cells depends on the inherent sensitivity of the tumor to chemotherapy, as well as the dose, frequency of administration, and the drugs route of entry into the tumor while ensuring therapeutic concentration is maintained. Despite having had extensive efforts taken during the past decade to add cytotoxic drugs as adjuvants to the Fu-nCRT regimen for LARC patients to improve the short- and long-term efficacy of neoadjuvant therapy, the benefits of these drugs being administered to patients are still not entirely adequate. The predominant drugs currently in clinical use are described below.

### Oxaliplatin

Oxaliplatin (Oxa) is a third-generation platinum-based chemotherapeutic drug that acts on DNA through the alkylated conjugates formed *in vivo* causing cross-chain and intra-chain cross-linking, leading to DNA damage and anticancer effects. Applicable diseases currently approved for Oxa are as follows: stage III (Duke's C) colorectal cancer after complete resection of primary tumor, metastatic colorectal cancer, and local advanced or metastatic hepatocellular carcinoma not suitable for surgical resection or local treatment. Furthermore, Oxa is usually combined with fluorouracil in clinical practice. Its common adverse reactions included marrow suppression, gastrointestinal reactions, and acute neurosensory symptoms.

Certain studies have shown that Oxa can enhance the sensitivity of tumor cells to radiation by inducing G2/M phase arrest and blocking DNA repair of tumor cells 12,13. In the past decade, in order to improve the efficacy of neoadjuvant therapy for LARC patients, a large number of studies have been conducted to add Oxa or an Oxa-containing chemotherapy regimen as an adjuvant to the Fu-nCRT regimen. Early studies on adding Oxa to the Fu-nCRT protocol found that incorporating the drug increased the pCR rate of patients, with the highest rate reaching 26% 14. However, among the researches the greatest number of patients enrolled was 58 15, and the least was 21 14. Given that the sample size is insignificant, these results are not well represented.

From 2010 to 2015, four large samples (598-1608 cases) of phase III clinical research results showed that, whether or not Oxa was used, the pCR rate of LARC patients did not exceed 20% 16-20. Only the German CAO/ARO/AIO-04 studies have shown that compared with the Fu-nCRT, the increased rate of PCR in patients using Oxa was statistically significant (13% vs.17%, *P* = 0.038) 19. Additionally, in terms of surgical results, The ACCORD (Actions Concertées dans les Cancers Colorectaux et Digestifs) 12 trial found, with or without Oxa, there was a significant difference in the positive rate of circumferential resection margin (CRM) ranging from 1-2 mm (9.9% vs. 19.3%, *P*=0.022) and no significant difference within 1 mm 16. Furthermore, The STAR (Studio Terapia Adiuvante Retto) 01 trial found that the addition of Oxa can significantly reduce the proportion of patients with intra-operative abdominal metastasis (0.5% vs. 2.9%, *P*=0.014) 17. In terms of early toxicity, the above four studies have all suggested that the addition of Oxa significantly increased the toxicity of nCRT, among which grade 3-4 diarrhea was the most obvious, with the incidence of 12%-25.4% in the Oxa group and 4%-10.9% in the control group. In addition, the ACCORD12 trial assessed the late toxicity of patients after 3 years of treatment, and found no difference in bowel control, erectile function, and social function in patients with or without Oxa 21. For the long-term efficacy, CAO/ARO/AIO-04 results showed that the 3-year disease-free survival (DFS) was 75.9% in the Oxa group and 71.2% in the control group (*P*=0.03). However, the 3-year overall survival (OS) and local recurrences rate (LRR) of the patients were 88% and 88.7%, 4.6% and 2.9%, respectively, with no statistical significance 20. According to the ACCORD 12 trial, the 3-year OS, LRR, and disease-free survival (DFS), with or without Oxa, were 87.6% and 88.3%, 6.1% and 4.4%, 67.9% and 72.7%, respectively, with no statistical difference 21.

Although only the CAO/ARO/AIO-04 study from Germany showed the benefit of pCR in the above sample study, the meta-analysis results of the four studies conducted by An et al. 22 in 2013 showed that the absolute difference of the pCR rate between the two groups were 2.5% (*P*=0.04), and the absolute difference of perioperative metastasis rate was 2.5% (*P*=0.001). The analysis of toxicity of patients showed that the incidence of 3/4 grade toxicity in the Oxa group was significantly increased, with an absolute difference of 8.7% (*P* = 0.004). In terms of treatment compliance, the radiotherapy prescription dose completion rate of the Oxa group was significantly lower than that of the control group, with an absolute difference of 8.0% (*P* = 0.004). Based on these data, Oxa is not currently recommended by the guidelines for inclusion in LARC nCRT regiments.

In spite of this, researchers are still exploring, but most clinical studies have shown that the addition of Oxa not only does not help to improve the pCR rate of patients^23^-^26^, but also significantly increases the incidence of therapeutic toxicity such as bone marrow suppression and diarrhea 23,25,26. In contrast, a meta-analysis of three large samples from 2017 to 2019 showed that although regimens using Oxa can expose patients to greater therapeutic toxicity, it is, however, beneficial for the short/long-term effect of patients 27-29. For example, Chen et al. conducted a network meta-analysis of 5599 patients in 14 randomized studies published prior to December 28, 2017, and found that the pCR rate of Oxa+Capecitabine (CAP) combined with radiotherapy (RT) was significantly higher than that of patients with 5-FU+RT. When analyzing with pCR rate as the primary target, it was found that Oxa+CAP+RT and CAP+RT were rated as the most effective and second effective regimens, respectively. In terms of treatment toxicity, the incidence of side effects of Oxa+5-FU+RT and Oxa+CAP+RT regimens was significantly higher than that of 5-FU/CAP+RT nCRT regimens. The incidence of treatment toxicity of 5-FU+RT and CAP+RT regimens were the lowest and the second lowest, respectively 29. In another meta-analysis, Zheng et al. analyzed 11 articles and associated ASCO (American Society of Clinical Oncology) abstracts from 8 studies, a total of 5597 cases, finding that adding Oxa to the Fu-nCRT can increase the pCR rate, extend DFS, and reduce both LRR and the distant metastasis rate (DMR), yet significantly increasing the therapeutic toxicity (RR=1.858, 95% CI: 1.427-2.419, *P*=0.000) 28. In the meta-analysis of prognosis of 3310 patients, De et al. found that whether or not Oxa was added had no significant effect on LRR, DFS, and OS, although adding Oxa had a significant effect on DMR reduction 27.

In view of the fact that ideal results were not achieved by adding the single drug Oxa as an adjuvant to a Fu-nCRT regimen, researchers carried out the exploration of total neoadjuvant therapy (TNT) which combines preoperative induction chemotherapy or consolidation chemotherapy with nCRT. The theoretical basis of this treatment model is that preoperative systemic chemotherapy can prevent or precociously eliminate the occult micrometastasis, thereby improving the local and systemic control rate and tumor resection rate 30. At present, FOLFOX (Oxa+leucovorin+5-Fu) and CAPOX (Oxa+CAP) are primary chemotherapy regimens in TNT with no uniform standard for chemotherapy cycle. The current studies show that TNT has advantages in the short-term efficacy and long-term prognosis of patients compared with Fu-nCRT. Cercek et al. found that 23 (53.5%) of the 43 stage II patients and 87 (32.8%) of the 265 stage III patients who received induction chemotherapy+nCRT obtained CR (pCR+cCR) 31. A prospective multi-center phase II trial by Julio et al. 32 investigated the efficacy of 0, 2, 4, or 6 cycles of FOLFOX added after Fu-nCRT and before TME surgery, and found that the pCR of patients were respectively 18%, 25%, 30%, and 38%. The odds ratio between the 6 cycles chemotherapy group and the single nCRT group was 3.49 (95% CI: 1.39-8.75; *P*=0.011). Pelvic abscess and anastomotic leakage were the most common complications and there was no statistical difference among groups. During the operation, it was found that compared with the nCRT group, the pelvic fibrosis of the patients treated with TNT was significantly increased, but with no significant difference in the technical difficulty of the operation and in intraoperative blood loss. The subsequent follow-up 33 with a median time of 59 months (range, 9 to 125 months) found that the DFS of the single nCRT group was significantly lower than that of the TNT group, and there was no significant difference in the DFS between the internal subgroups of the TNT group. Cox regression model found that whether TNT was carried out was one of the independent factors affecting DFS, and there was no significant difference in OS between the single nCRT group and the TNT groups. Petrelli et al. 34 conducted a meta-analysis on the short/long-term efficacy of 3591 patients in 28 studies and found that the pCR rate of TNT regimen was 22% (the odds ratio was 39% higher than the Fu-nCRT), and the treatment completion rate was 81.9% to 100%. Treatment-related toxicity mainly included radioactive dermatitis, diarrhea, proctitis and hematological toxicity; all of which were similar to that of Fu-nCRT. Patients treated with TNT had significantly higher OS than those treated with Fu-nCRT (HR=0.73, 95%CI 0.59-0.9, *P*=0.004), and better results were obtained in DFS, but no statistical difference was found when compared with Fu-nCRT.

From the above results, we found that adding single drug Oxa to the Fu-nCRT regimen, recommended by the current guidelines, seems to show advantages in increasing pCR rates and reducing LRR and DMR, but it also brings forth the risk of increased toxicity and decreased treatment compliance, which may also be the reason that the short-term efficacy advantage of Oxa+nCRT has not been transformed into a long-term survival advantage. Although TNT therapy has achieved more optimistic results than those of Fu-nCRT in clinical trials, data from large randomized studies and long-term follow-up are still limited, so this treatment model was only mentioned in the guidelines as a treatment strategy for LARC patients.

### Irinotecan

Irinotecan (CPT-11), a plant-based water-soluble camptothecin precursor chemotherapeutic drug, is a selective DNA topoisomerase I (Topo I) inhibitor, which can be converted into 7-ethyl-10-hydroxycamptothecin (SN38) *in vivo*. SN38 covalently combines with DNA topoisomerase-DNA complex to form DNA topoisomerase-drug-DNA complex in cells, inhibiting the activity of Topoi, thus interfering with the reconnection of the DNA single-strands of tumor cells. This leads to DNA strand breakage, promotes tumor cell apoptosis, and has the greatest toxicity to cells in S phase 35. It is found that CPT-11 forms a stable CPT-11-TopoI-DNA complex between the attachment of the DNA single-strand break site and the topoi-dna complex, which plays a specific role in the S phase of a cell cycle. At present, the approved applicable disease of CPT-11 is advanced colorectal cancer. Single drug CPT-11 can be used for patients with advanced colorectal cancer who have previously shown low or no response to 5-FU chemotherapy. For patients with advanced colorectal cancer who have previously received chemotherapy, CPT-11 is usually combined with 5-FU and leucovorin to form the FOLFIRI regimen. The common adverse reactions of this drug included marrow suppression and gastrointestinal reactions mainly manifested by delayed diarrhea. Studies have found that radiation not only can cause DNA strand breakage, but can also cause tumor cells to regroup, which in turn causes most tumor cells to remain in S phase, increasing patient's sensitivity to CPT-11 36. Therefore, theoretically, there is a synergistic relationship between CPT-11 and irradiation.

The current results of CPT-11 participating in nCRT of LARC show that the pCR rate obtained by patients in different studies are quite different, approximately 10%-34.7% 37-42. Stuart et al's RTOG 0247 study 38 showed that the pCR rates of CPT-11+CAP+RT and Oxa+CAP+RT were 10% and 21%, respectively, and the CPT-11 group was significantly lower than the Oxa Group. However, Sato's single-arm study showed that the effective rate of CPT-11+S1+RT was 68.7%, and the pCR rate was 34.7% 39. In terms of long-term efficacy, Gollins et al's single-arm study showed that CPT-11+CAP+RT treatment had 3-year local recurrence-free survival (LRFS), distance metastasis-free survival (DMFS), DFS, and OS which were 96.9%, 71.1%, 63.5% and 88.2%, respectively. The pCR+proximity-pCR rate was an independent factor affecting 3-year OS with no significant difference between the pCR rate and proximity-pCR rate on OS. The pCR+proximity-pCR rate and postoperative lymph node stage were independent factors affecting DFS. According to these results, researchers believe that the recent high response of tumors to CPT-11+CAP+RT treatment is an important marker for patients to obtain a longer survival period 40. However, Sung et al. found no difference in local control (LC), recurrence-free survival (RFS), and OS between the two groups by comparing the efficacy of CAP+RT and CPT-11+CAP+RT 43. A follow-up study of RTOG 0247 44 also found that the 4-year DFS, OS, LRF, and DMF of patients adding CAP-11 or Oxa with CAP+nCRT were 68%, 85%, 16%, 24% and 62%, 75%, 18% and 30%, respectively, with no statistical difference. After comprehensive analysis of the results of the two stages 38,44, researchers concluded that the low 10% pCR rate obtained by CAP-11 combined with CAP+nCRT does not translate into a poor prognosis, therefore the use of pCR rate as a survival indicator for patients remains to be explored.

In terms of treatment-related toxicity, multiple studies found that the addition of CPT-11 significantly increased the risk of myelosuppression and diarrhea in patients 37, 45-46. CPT-11 can be converted into the more cytotoxic active metabolite SN-38 *in vivo* and then inactivated by uridine diphosphate (UDP)-glucuronosyltransferases (UGTs) in the liver to SN-38 glucuronide (SN-38G), which is converted into SN-38 through bile in the small intestines under the action of β-glucuronidase, which then leads to drug toxicity dominated by granulocytopenia and diarrhea 47, 48. UGT1A1, UDP-glucuronosyltransferases family 1 member A1 (human), is an important metabolic enzyme of CPT-11. Studies have found that there is a large number of T/A base repeats in the promoter region of UGT1A1. With the increased number of T/A repeats, the expression of UGT1A1 will decrease, which will lead to the excessive accumulation of SN-38, causing severe CPT-11 toxicity 48.

At present, there are a limited amount of reports about UGT1A1 genotype and CPT-11 participating in nCRT of LARC patients. We only retrieved two studies in this field on the United States National Library of Medicine National Institutes of Health website, among which Kimura results 49 showed that the polymorphism of UGT1A1 affected the incidence of neutropenia. The level 3-4 neutropenia and leucopenia in heterozygote and homozygote groups were significantly higher than those in the wild-type group, but the effect on diarrhea was not found. Due to the purpose of this study, the relationship between short-term and long-term efficacy and UGT1A1 gene polymorphism was not analyzed.

In another report 50, the maximum tolerable dose (MTD) of CPT-11 in CPT-11+ Fu-nCRT regimens in patients with UGT1A1*1 and UGT1A1*28 genotype LARC were analyzed. It was found that the level 3-4 toxicity was mainly neutropenia and diarrhea, and there was a correlation between the incidence and the dose of CPT-11. Finally, it was found that the MTD of patients with UGT1A1*1 genotype and UGT1A1*28 genotype were 80 mg/m2 and 65 mg/m2, respectively. In a follow-up report 51, researchers analyzed the therapeutic toxicity, pCR rate, treatment compliance, and DFS of 52 LARC patients with UGT1A1*1 genotype, and found that 38.46% of the patients had grade 3/4 toxicity (including leukocytosis (21.15%), neutrophils (19.23%), diarrhea (23.08%)), 23.08% pCR rate, 7.69% clinical complete remission (cCR) rate, 80.77% treatment compliance rate, and 64.1% 3-year DFS. Therefore, the researchers believe that CPT-11 can achieve encouraging short-term efficacy (pCR + cCR=30.77%) in patients with UGT1A1*1 genotype LRAC, although its therapeutic toxicity is high, it is still within controllable range.

In summary, there is currently a large difference in the reports on the pCR rate obtained by CPT-11 combined with Fu-nCRT. The data based on the UGT1A1 genotype and the patients' short-term/long-term efficacy and treatment-related toxicity are still limited, and a large sample size randomized controlled study still needs to be carried out.

## Molecular targeted anticancer drugs

Molecular targeted anticancer drugs are designed for specific gene mutation targets at the cellular and molecular level. After entering the body, the drugs can specifically combine with the target, leading to the death of tumor cells. Prior to administering, the patients need to be gene sequenced and treatment be individualized based on the target. These drugs are mainly divided into monoclonal antibodies and small molecule kinase inhibitors. At present, molecular targeted anticancer drugs have been widely used in the treatment of colorectal cancer, and numerous explorationshave been carried out in the field of neoadjuvant therapy for LARC patients. The introduction is as follows:

### Bevacizumab

Bevacizumab is a recombinant humanized IgG1 monoclonal antibody with 214 amino acids and a molecular weight of 149000 Dalton. It can selectively bind to VEGF and inhibit the binding of VEGF to FLT-1 and KDR on the surface of endothelial cells, resulting in the inability of endogenous VEGF to exert its biological activity, thus inhibiting the proliferation and migration of vascular endothelial cells, inducing endothelial cell apoptosis, inhibiting the formation of tumor angiogenesis, and promoting the existing abnormal regression vessels 52-53. Currently, bevacizumab is approved for use in combination with 5-FU for metastatic colorectal cancer and platinum-based chemotherapy regimens for unresectable metastatic or recurrent non-squamous non-small cell lung cancer. It was found that the main therapeutic toxicities of bevacizumab include hypertension, bleeding, thrombotic events, and wound healing disorders among others. There is no significant overlap with the toxicity of chemotherapy drugs 54-56.

Previous studies have found that radiation-induced VEGF can increase the radiation resistance of tumors, while the treatment of anti-VEGF can reduce the radiation resistance of tumors 57-59. At present, there is a large amount of evidence determining the efficacy of bevacizumab in the systematic treatment of colorectal cancer patients, but its application in nCRT for LARC patients is still being explored. Existing research 60-66 shows that adding bevacizumab to the Fu-nCRT regimen can achieve a pCR rate of 16-36% for LARC patients, compared to the 12%-34% obtained by Oxa+Fu-nCRT regimen 14-19, 23-26, and is numerically superior to the 11%-15% achieved by the independent Fu-nCRT regimen 9, 10. In the phase II study of Xiao et al. 67, 25 patients with LARC were treated with sandwich-like neoadjuvant therapy, that is, bevacizumab combined with FOLFOX induction + Fu-nCRT + sequential FOLFOX. Surgical resection performed 4-6 weeks after revealed that 18 patients (72%) obtained pathological remission and 7 (28%) were in stable condition. Of the 25 patients, 23 patients (92%) received surgical treatment and 2 patients (8%) refused resection due to personal reasons. The patients' pCR was 39.1% (9/23), and 3-year DFS and OS were 72.5% and 95%, respectively.

However, most researchers are more concerned about the increased risk of postoperative complications, especially when satisfied with the high PCR rates obtained by adding bevacizumab to nCRT. Dellas et al. 68 analyzed the postoperative complications of 62 LARC patients who received concurrent preoperative bevacizumab+Oxa+CAP-RT treatment and found that the incidence of anastomotic leakage was 17.5% (7 cases) in 40 patients with anterior/low anterior resection, incision infection rate was 6.1% (3 cases) in 49 patients with abdominal resection, and perineal incision infection rate was 25% (5 cases) in 20 patients with abdominoperineal resection. Among the enrolled patients, 8 (12.9%) experienced delayed wound healing, 3 (4.8%) had abdominal/presacral abscesses, and 43.5% had postoperative complications, with the average duration of surgery being 239 min (±10). Liang et al. 69 evaluated the technical feasibility of laparoscopic TME after neoadjuvant treatment (bevacizumab+FOLFOX-nCRT) and found that the average duration of surgery was 214.4 min (± 44.4), the incidence of complications such as upper gastrointestinal hemorrhage, deep vein thrombosis, pelvic abscess, wound infection, external intestinal fistula and perineum fistula were 21.4%. Of those same patients, 78.6% achieved adequate pathological response with a pCR rate of 25%, and a tumor cell microresidue (<10% of the microscopic field of vision) rate of 53.6%. Comparing the above two studies, it was found that the incidence of postoperative complications was significantly lower in Liang's patients than those of Dellas' (21.4%vs. 43.5%). We believe that the reason may be related to the adoption of less invasive laparoscopic surgery in Liang's patients. In another report, the researchers adopted a neoadjuvant treatment regimen which was more aggressive than that of Dellas' and Liang's neoadjuvant combination. Prior to the initiation of bevacizumab+5-Fu-nCRT, they carried out induction therapy using bevacizumab+FOLFOX6. During the treatment period, 76% of patients had grade 3/4 toxicity, of which diarrhea, neutropenia, and pain were the most common, leading to discontinuation of the study; follow-up observation found that 36% of the patients experienced postoperative complications 70.

In addition, we also obtained a number of case reports on a total of 10 patients concerning delayed anastomotic fistula caused by bevacizumab+nCRT 71-77. The interval between operation and anastomotic leakage was 17-60 months in these patients, among which 5 patients had previously experienced anastomotic leakage; 4 of them had used more than one course of bevacizumab and among the remaining patients who experienced anastomotic leakage for the first time, 3 years had been the longest course of bevacizumab. Although this delayed complication is relatively rare, its consequences are quite serious, therefore, we believe that special attention should be paid to the long-term postoperative complications caused by bevacizumab +nCRT.

In summary, adding bevacizumab to nCRT can greatly improve the PCR rate of patients, but the increase of postoperative complications has become the point of concern for clinical promotion. Furthermore, Laparoscopic surgery may be a feasible solution, but for patients who strongly oppose surgery, Bevacizumab combined with CRT is likely to be a better option.

### Cetuximab

Cetuximab is an IgG1 monoclonal antibody targeting EGFR. It can specifically bind to the extracellular domain of EGFR, block the binding of natural ligand and EGFR receptor, inhibit signal transduction, and promote the internalization and degradation of EGFR receptor. In addition, cetuximab can induce antibody dependent cell-mediated cytotoxicity, thereby playing a role in inhibiting and killing tumor cells 78. RAS gene is one of the important downstream signal molecules of the EGFR-related signal transduction pathway. Previous studies have confirmed that RAS is a positive molecular predictor for primary resistance to anti-EGFR therapy 79, 80. The clinical efficacy of cetuximab has been confirmed to be closely related to the status of RAS (esp. KRAS and NRAS). Currently, it was approved for use in combination with FOLFOX or FOLFIRI chemotherapy regimen for the advanced colorectal cancer patients with wild-type RAS gene. The significant adverse reactions due to therapeutic toxicity associated with cetuximab are mainly skin reaction and diarrhea.

In recent years, in order to improve treatment results, researchers have tried to add cetuximab to nCRT treatment of LARC patients. For example, in 2012, EXPERT-C study 81 enrolled 165 LARC patients, 90 of whom were KRAS and/or BRAF wild-type patients. The neoadjuvant treatment regimen was divided into four stages: CAPOX (4 cycles)+ CAP-nCRT+ TME+ CAPOX, patients were divided into two groups with and without cetuximab at the beginning of CAP-nCRT. At the end of each stage, the efficacy was evaluated by computerized tomography (CT) and magnetic resonance imaging (MRI). The results showed that there was no difference in LRR, PFS, and OS between the two groups without genotype differentiation. In 90 patients with genotype wild-type, the local remission rate (CR+PR) with and without cetuximab were 93% and 75%, respectively (*P*=0.028) and pCR rates were 11% and 7%, respectively (*P*=0.74). There was no difference in 5-year PFS but significant difference in 5-year OS (*P*=0.034). Although the CR+PR and OS of genotype wild-type patients in the EXPERT-C study were superior to those in the control group, the 11% pCR rate did not seem to provide a benefit for the choice of follow-up treatment. In contrast, Gollins et al. found that the favorable clinical/pathological response rate of cetuximab was 48% in patients with RAS wild-type and 20% in the mutant group, but there was no significant difference in PFS and OS between the two groups 82. In SWOG 0713 study of Leichman et al., investigators conducted a single-arm study on 83 patients with KRAS wild-type, one cycle of induction therapy with Oxa+CAP+cetuximab followed by cetuximab+CAP-nCRT. The pCR rate was 27%, and the 3-year DFS was 72%, but more than 10% of the patients suffered grade 3 or higher treatment toxicity, such as diarrhea, skin rash, electrolyte disorder, including 1 case treatment-related death from multiple organ failure. Although the experiment achieved a pCR rate of 27%, it did not meet the benefit requirements of SWOG regarding the addition of new drugs to existing treatment regimens 83, so the researchers believe that there is insufficient clinical evidence to recommend the addition of cetuximab to patients with KRAS wild-type LARC in Fu-nCRT regimen 84.

For the patients with KRAS mutation, Cuneo et al. conducted a cell experiment and found that the application of crizotinib, a tyrosine kinase inhibitor (TKI), targeting c-Met gene expression could shorten the stagnation of tumor cells in G1 phase, and block hepatocyte growth factor (HGF). HGF, the main ligand of c-Met, induces cell migration and enhances the radiosensitivity of KRAS mutant colorectal cancer cells 85. In addition, during our literature search, we found that no relevant studies were reported on the BRAF-V600E mutation inhibitors vemurafenib, dabrafenib, and trametinib in nCRT of LARC patients.

In short, the current research shows that adding cetuximab to the Fu-nCRT regimen cannot benefit the patients without distinguishing the genotypes, and the benefit rate of adding cetuximab to the gene wild-type patients seems unclear, so the clinical value of adding cetuximab to the Fu-nCRT regimen still needs more research data to support.

### Panitumumab

Panitumumab is a fully humanized IgG2 monoclonal antibody with a high affinity for EGFR. By binding to EGFR specifically, panitumumab inhibits the binding of EGFR to its ligand, thus preventing ligand-induced autophosphorylation of the receptor and activation of receptor-related kinases, thereby inhibiting tumor growth and inducing apoptosis of cancer cells. In other words, panitumumab and cetuximab have similar anticancer mechanisms, but the risk of drug toxicity is much lower than that of cetuximab 86. A number of studies have shown that panitumumab combined with chemotherapy has good efficacy and safety in first-line, second-line, and third-line treatment of patients with wild type KRAS advanced colorectal cancer 87-89.

At present, the results of panitumumab applied to nCRT in LRAC patients show that the addition of panitumumab cannot improve the clinical efficacy, which is particularly obvious in the study of panitumumab combined with single-dose radiotherapy. For example: one single-arm phase II study conducted by Merx et al. found that the combination of panitumumab and radiotherapy in patients with RAS wild-type LARC only achieved a pCR rate of 3.7%, with skin toxicities and incidence of diarrhea above grade 3 being 24% and 10%, respectively 90. In the RaP Study/STAR-03 study conducted by Pinto et al., panitumumab combined with radiotherapy achieved a pCR rate of 10.9% in KRAS wild-type LARC patients, and a grade 3 skin toxicity of 16.3% 91. Similarly, the combination of panitumumab and nCRT did not show obvious advantages. For example, in the StarPan/star-02 study, researchers added panitumumab to the Oxa+ Fu-nCRT regimen and obtained a pCR rate of 22.1%, but the grade 3/4 diarrhea of patients reached 38.9%, and one patient suffered toxic-related death 92. In SAKK 41/07 study 93, the pathological near-complete response (pNCR)+ pCR rate obtained by adding panitumumab to Fu-nCRT of KRAS wild-type patients was 53%, and the actual pCR rate was only 10%, which was 32% and 18% in the control group, respectively. In patients with pNCR+pCR, the incidence of diarrhea was 10% in the panitumumab group and 6% in the control group; the incidence of postoperative anastomotic fistula was 15% and 4% in both groups. Therefore, researchers believe that the addition of panitumumab in Fu-nCRT regimen brought the patients non-negligible therapeutic toxicity.

In summary, the current study shows that the addition of panitumumab to the nCRT regimen not only does not bring gratifying clinical and pathological effects to patients, but also increases the patient's therapeutic toxicity and the risk of high postoperative complications.

### Gefitinib

Gefitinib is a selective EGFR tyrosine kinase inhibitor. It can block the signal transduction pathway of proliferation, growth, and survival of cancer cells by inhibiting the activity of EGFR tyrosine kinase and promoting the apoptosis of cancer cells while preventing tumor growth. It is used for the treatment of locally advanced or metastatic non-small cell lung cancer (NSCLC) patients with sensitive mutations of EGFR. Skin rash, interstitial pulmonary fibrosis and diarrhea are the main adverse drug reactions. Currently, there are a small number of reports on the application of gefitinib in neoadjuvant therapy for LARC patients. *In vitro* studies by Palumbo et al. found that gefitinib can promote the accumulation of rectal cancer LoVo cells in G1 phase, reduce the proliferation of 5-Fu and 5-Fu+RT surviving cells, and increase the cytotoxicity of RT and 5-Fu 94. However, the addition of Gefitinib in the Fu-nCRT did not achieve ideal results in the clinical study. Czito et al. 95 found that in the 6 LRAC patients, there were 0 cases of pCR, 1 case of disease progression, and 2 cases of dose limited toxicity. Valentini's study 96 showed that after the addition of gefitinib in the 5-Fu-nCRT regimen, patients obtained 30.3% (10/33) pCR rate, but the incidence of toxicity above grade 3 was up to 41%, among which the incidence of gastrointestinal toxicity was 20.5%, skin toxicity was 15.3%, and genitourinary toxicity was 10.2%. Follow-up reports showed no significant advantage in the prognosis of patients compared with other literature data, but 38.4% of patients developed grade 3-4 delayed toxicity including sexual dysfunction (28.2%) and gastrointestinal toxicity (10.2%) 97.

Concisely, the current study shows that the addition of gefitinib to Fu-nCRT does not significantly improve the efficacy of patients, and the near and long term treatment toxicity of patients are obvious. However, these studies were conducted without clarifying the genetic mutations in the enrolled patients, so further studies on Gefitinib+Fu-nCRT regimen in LARC patients with specific EGFR status still need to be carried out.

## Immune checkpoint inhibitors

Current research shows that immunotherapy targeting cytotoxic T-lymphocyte antigen 4 (CTLA-4), programmed cell death protein 1 (PD-1) and programmed death ligand-1 (PD-L1) can significantly improve patient survival of metastatic melanoma, urothelial carcinoma, prostate cancer, non-small cell lung cancer, and other malignant tumors 98-101. So far, the drugs targeting CTLA-4 approved by FDA are ipilimumab, targeting PD-1 are nivolumab and pembrolizumab, and targeting PDL-1 include atezolizumab, durvalumab, avelumab, and cemiplimab-rwlc. Among them, ipilimumab and nivolumab were approved for colorectal cancer. The adverse drugs reactions of immune checkpoint inhibitors mainly include gastrointestinal toxicity characterized by diarrhea, skin toxicity characterized byrash, pulmonary toxicity characterized by fibrosis, hepatotoxicity characterized by elevated transaminase, hematological toxicity characterized by thrombocytopenia and neutropenia, endocrine toxicity characterized by thyroid dysfunction, and immune myocarditis that are rare in clinical practice, and so on 102, 103. Several studies have shown that radiotherapy can not only improve the oxygenation level and PH value of tumor cells, up-regulate the expression of cell adhesion molecules, promote the reconstruction of extracellular matrix and tumor blood vessels, but can also enhance the recruitment of tumor immune effector cells and their response to immunotherapy 104-106.

Microsatellite instability (MSI) is an important molecular marker closely related to the biological behavior of tumor and the therapeutic effect of immunotherapy caused by the defect of mis-match repair gene. In 2020, Hasan et al. reported the results of multivariate regression analysis of MSI status, nCRT response, and prognosis of 5086 LARC patients in the national cancer database (NCDB), affirming that the pCR rates of MSI (-) patients and MSI (+) patients were significantly different, 8.9% and 5.9%, respectively. MSI(+) is an independent factor that leads to low pCR rates in LARC patients after receiving nCRT, and researchers believe that increasing attention to MSI status may help clinicians recommend more suitable neoadjuvant treatment for patients 107. In another study, Tominaga et al. evaluated the changes of serum PD-1 and PD-L1 in patients with low LARC before and after nCRT, as well as the relationship between these changes, pathological features, and DFS. It was found that the PD-L1 substantially increased after nCRT, which was closely correlated with the vascular invasion of the tumor and somewhat correlated with inadequate DFS (P=0.075). Therefore, the researchers believed that the increased serum PD-L1 level after nCRT indicated the combination of immunotherapy for PD-L1 and nCRT might be a potential treatment strategy for LARC patients 108. At present, there are no reports on the research of adding tumor immune checkpoint inhibitors to nCRT. Zhang et al. 109 reported the results of neoadjuvant treatment of 2 cases of LARC with cT4N2M0 by using nivolumab alone; all patients had high MSI expression. Immunofluorescence staining showed that PD-L1 positive tumor cells, CD68(+) macrophages, and CD8(+) T cells were present in the samples before treatment; after treatment, a large influx of CD8(+) T cells and high expression of PD-L1 in immune cells were observed. The pCR was obtained in both patients after 6 courses of single drug nivolumab neoadjuvant therapy.

In summary, at present, the mechanism of promoting tumor immunotherapy response by radiotherapy has been relatively clear, and the application of immune checkpoint inhibitors alone in preoperative neoadjuvant therapy for LARC patients have seem to become apparent. It is expected that future research combined with nCRT may be beneficial to patients.

## Conclusion and Expectation

At present, nCRT, as an important part of standard treatment for LARC patients, has been widely used in clinical practice. The addition of cytotoxic drugs and Bevacizumab may bring higher pCR rates for patients, but the research on the reduction of therapeutic toxicity and postoperative complications remains to be actively carried out. The clinical application of TNT has achieved satisfactory results in short-term efficacy, however, there are still limited amounts of large sample randomized controlled trials and follow-up results. The data obtained from the addition of EGFR inhibitors in the Fu-nCRT regimen are not optimistic, and there is still much room for research of the precise treatment based on gene phenotype. The application of immune checkpoint inhibitors in patients receiving radiotherapy has some theoretical basis, but still lacks the support of clinical data. We speculate that in the future, according to the results of immunoassay points and related markers, the combination of immunotherapy and nCRT in LARC patients may become a new point of interest.
